# Whole-Genome Analysis of *Aeromonas hydrophila* Strain 187, Exhibiting Quorum-Sensing Activity

**DOI:** 10.1128/genomeA.01360-14

**Published:** 2014-12-24

**Authors:** Xin-Yue Chan, Kek Heng Chua, Wai-Fong Yin, S. D. Puthucheary, Kok-Gan Chan

**Affiliations:** a Division of Genetics and Molecular Biology, Institute of Biological Sciences, Faculty of Science, University of Malaya, Kuala Lumpur, Malaysia; b Department of Biomedical Science, Faculty of Medicine, University of Malaya, Kuala Lumpur, Malaysia; c Department of Medical Education, Research and Evaluation, Duke-NUS Graduate Medical School Singapore, Singapore

## Abstract

*Aeromonas hydrophila* is a quorum-sensing (QS) bacterium that causes diarrhea in humans upon infection. Here, we report the genome of pathogenic *Aeromonas hydrophila* strain 187, which possesses a QS gene responsible for signaling molecule *N*-acyl homoserine lactone (AHL) synthesis and has been found to be located at contig 36.

## GENOME ANNOUNCEMENT

*Aeromonas hydrophila* is a quorum-sensing (QS) bacterium that causes human gastrointestinal and extra-intestinal diseases (1, 2). The expressions of its virulence factor, including the exoprotease, biofilm formation, and hemolysin coregulated protein, are QS regulated (3–5). In a previous study, the QS signaling molecule *N*-acyl homoserine lactone (AHL) profile of *A. hydrophila* 187 was characterized. However, the gene coding for its AHL synthase remains unknown. In this study, we report the whole genome and QS gene of *A. hydrophila* 187.

*A. hydrophila* strain 187 was isolated from a patient’s pus at the University Hospital, University of Malaya, Kuala Lumpur, Malaysia (6). The isolate was maintained using LB media at 37°C. Genomic DNA of the *A. hydrophila* strain was extracted using a QIAamp DNA MiniKit (Qiagen, Germany) (7). Subsequently, the DNA was quantified and qualified using a Qubit 2.0 fluorometer (Invitrogen, USA) and Nanodrop (Thermo Scientific, USA) prior to next generation sequencing library preparation with a Nextera DNA sample preparation kit (Illumina, USA). The whole-genome sequencing was performed using MiSeq (Illumina, USA) (8). Paired-end reads were trimmed and *de novo* assembled with CLC genomic workbench v5.1 (9). The contigs were subjected to gene prediction with Prodigal followed by gene annotation by BLAST against the Uniprot database (10, 11).

A total of 3.48 million reads were generated from this whole-genome sequencing. After quality trimming, the reads were assembled into 59 contigs, with an average coverage of 111-fold and *N*_50_ of 197 kbp. The draft genome of *A. hydrophila* strain 187 is 4.7 Mbp and the G+C content of its genome is 61.63%. Gene prediction has identified 4,339 coding DNA sequences (CDS) from the genome.

Based on the annotation result, *luxI* homolog (*ahyI*) of *A. hydrophila* 187 was detected in contig 37. The length of this gene is 621 bp located at 45,853 bp to 48,473 bp of contig 37. The *ahyI* gene encodes for *A. hydrophila* AHL synthase, which is responsible for the production of its QS signaling molecule (12). Recently, another member of *Aeromonas* that exhibits QS properties, *A. caviae*, has been isolated from garden compost samples (13). With the availability of this genome sequence, further characterization of the *A. hydrophila* strain 187 could be carried out using our whole-genome data, which could lead to an understanding of the relation of QS and the virulence factor of this bacterium.

### Nucleotide sequence accession numbers.

This draft genome was deposited into DDBJ/EMBL/GenBank under the accession no. AOBO00000000. The version described in this paper is the first version, AOBO01000000.
